# The Potential Roles of Actin in The Nucleus

**DOI:** 10.22074/cellj.2015.507

**Published:** 2015-04-08

**Authors:** Khadijeh Falahzadeh, Amir Banaei-Esfahani, Maryam Shahhoseini

**Affiliations:** 1 Department of Genetics at Reproductive Biomedicine Research Center, Royan Institute for Reproductive Biomedicine, ACECR, Tehran, Iran; 2 Department of Cell and Molecular Biology, Faculty of Biological Sciences, Kharazmi University (TMU), Tehran, Iran; 3 Department of Biotechnology, College of Science, University of Tehran, Tehran, Iran

**Keywords:** Actin, Nuclear Matrix, Chromatin Remodeling, Transcription

## Abstract

Over the past few decades, actin’s presence in the nucleus has been demonstrated. Actin is a key protein necessary for different nuclear processes. Although actin is well known for its functional role in dynamic behavior of the cytoskeleton, emerging studies are now highlighting new roles for actin. At the present time there is no doubt about the presence of actin in the nucleus. A number of studies have uncovered the functional involvement of actin in nuclear processes. Actin as one of the nuclear components has its own structured and functional rules, such as nuclear matrix association, chromatin remodeling, transcription by RNA polymerases I, II, III and mRNA processing. In this historical review, we attempt to provide an overview of our current understanding of the functions of actin in the nucleus.

## Introduction

Actin, a globular protein with an approximately 42 kDa molecular weight, is found in all eukaryotic cells as one of the most highly-conserved proteins of the cytoskeleton. Although actin is often thought of as a single protein, in mammals it actually consists of six different isoforms encoded by distinct genes (1). All of the isoforms possess remarkably similar amino acid sequences, with no isoform sharing less than 93% identity with any other isoform. Recent studies indicate that actin isoforms carry out unique cellular functions. Four isoforms of actin including α-skeletal actin, α-cardiac actin, α-smooth actin and γ-smooth actin are expressed primarily in skeletal, cardiac, and smooth muscle, respectively. The remaining two isoforms, β-cyto actin and γ-cyto actin are basically expressed universally (2). 

The fundamental roles of actin are well-known in critical biological processes such as cell migration, determination of cell shape, and vesicle trafficking (3). While actin’s roles in the cytoskeleton and cytoplasm are well established, the presence of nuclear actin has been a controversial issue for many years, owing to the lack of definitive evidence (4). Recent evidence shows the association of actin with multiple nuclear complexes, thus the existence of actin in the nucleus is slowly being accepted. 

To date, actin has been implicated in various structured and functional roles in nuclears, including nuclear matrix association, chromatin remodeling, transcription and mRNA processing (5). Mass spectrometry (6) as well as immunoreactivity experiments (7,8) show that β-actin is the nuclear isoform of actin associated with heterogeneous nuclear ribonucleoproteins ( hnRNPs ) and RNA polymerase complexes. Schoenenberger et al. (9) have discovered that different polymerization states of actin co-exist in the nucleus. Studies of fluorescence recovery after photobleaching revealed that ~20% of the nuclear actin pool has characteristics of a very dynamic polymeric population (10). These results supported the existence of nuclear actin in polymeric form. It would be of interest to determine whether any of the functions of actin in the nucleus rely on polymerization/depolymerization events (3). 

## History of nuclear actin identification

The presence of actin in eukaryotic cell nuclei was originally reported by Ohnishi et al. (11) in 1963 when they purified actin from the nuclei of calf thymocytes. Historically, the studies that introduced actin as a nuclear protein were initially met with massive skepticism. For many years its presence in the nucleus was considered to be an artifact. The main problem was the inability to reliably detect actin protein in the nucleus by immunofluorescence microscopy (12). 

Most known functions of actin in the cytoplasm encompass polymerization of actin monomers ( globular actin or G-actin ) into actin polymers ( filamentous actin or F-actin ), which is key for the diverse functions of cytoskeletal actin.The most common method to detect actin filaments in the cytoplasm is phalloidin staining which detects actin filaments with at least seven actin monomers (13,14). However, under normal conditions, nuclei and consequently nuclear actin can not be stained by phalloidin. Therefore, the actin filaments could not be visualized in the cell nucleus in the manner that they were commonly observed in the cytoplasm (15). 

In 1977, Clark and Merriam (16) conclusively identified the existence of actin in the nuclei of a *Xenopus* oocyte by removing the nuclear envelope of manually isolated oocyte nuclei. By using a more refined method, Pederson extracted chromatin from *Dictyostelium discoideum* that was expected to have less than 0.3% cytoplasmic contamination in the purified chromatin fraction. He demonstrated that actin’s concentration in this chromatin fraction exceeded the cytoplasmic concentration by several orders of magnitude (17). Subsequently, actin was identified in mammalian cell nuclei by immunoelectron microscopic studies (12). 

Research experiments performed in the last decade have provided convincing evidence for the presence of actin in the cell nucleus and for the roles of actin in fundamental nuclear processes. A list of some organisms and cell types where actin has been identified is shown in table 1. 

**Table 1:** List of some species and cell types in which nuclear actin has been reported


Species	Cell types	References

**Protists**	*Physarum polycephalum*	Jockusch et al. (18)
	*Tetrahymena pyriformis*	Katsumaru and Fukui (19)
	*Dinoflagellate*	Soyer-Gobillard et al. (20)
**Plants**	*Sauromatum guttatum*	Skubatz et al. (21)
	*Allium cepa*	Cruz et al. (22)
**Insects**	*Spodoptera frugiperda*	Volkman (23)
	*Drosophila melanogaster*	Sauman and Berry (24)
**Amphibians**	*Xenopus laevis*	Merriam and Hill (25)
	*Rana temporaria*	Parfenov et al. (26)
**Birds**	*Duck erythroblasts*	Maundrell and Scherrer (27)
	*Chicken livers*	Crowley and Brasch (28)
**Mammalian cells**	*Thymus cells*	Ohnishi et al. (11)
	*Myoblasts*	Paulin et al. (29)
	*Ovary cells*	Brunel and Lelay (30)
	*Neural cells*	Sahlas et al. (31)


## Nucleocytoplasmic translocation of actin

The nucleocytoplasmic traffic occurs through nuclear pore complexes which form channels through the nuclear membrane. Two forms of transport occur over nuclear pores: I. passive diffusion of proteins smaller than 40 kDa and II. an active, energy-dependent traffic facilitated by specific transport receptors (32). The size of actin ( approximately 42 kDa ) is over the limit for passive diffusion through the nuclear pore. Therefore, there are some indications that actin may use this method to enter the nucleus (5). 

## Nuclear import of actin

Actin itself does not contain a classical nuclear localization signal ( NLS ), and until now no specific import receptor for actin has been reported. However, various actin-binding proteins ( ABPs ), such as cofilin (33), CapG (34) and megakaryocytic acute leukemia ( MAL ) (35) contain an NLS. Therefore these proteins can "piggy-back" actin into the nucleus. Cofilin is reported to be essential for nuclear accumulation of actin in response to cellular stress (36). 

According to Pendleton et al. (36) latrunculin B treatment in mast cells induced formation of intranuclear filament which can be inhibited by antibodies against cofilin. Nevertheless, it is currently uncertain whether cofilin or other ABPs facilitate active transport of actin into the nucleus under physiological conditions (12). Recently, it has been shown that importin 9 works as an import factor for nuclear actins (37). 

## Nuclear export of actin

**Fig.1:**
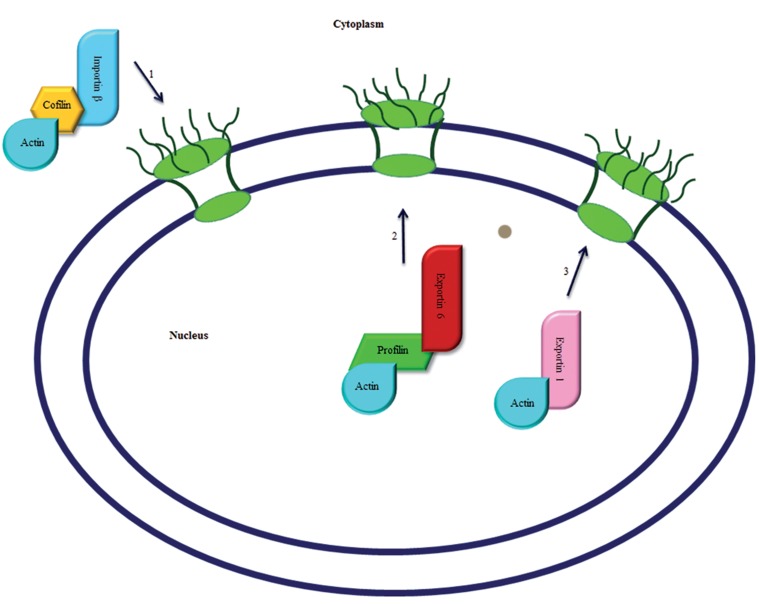
Nucleocytoplasmic translocation of actin. 1. Nuclear import of actin. Cofilin enters the nucleus through the import receptor, importin β. 2, 3. Nuclear export of actin. Actin can exit the nucleus in coupled with the actin binding protein, profilin, through the export receptor exportin 6 and/or through the exportin 1 receptor.

Actin appears to use an active transport mechanism to exit the nucleus. It is exported to the cytoplasm by at least two different pathways (Fig.1). Firstly, actin owns two classical leucine-rich nuclear export signals ( NES ), which exist in all of actin’s isoforms. These signals are essential for the export of actin via the export receptor exportin 1. Treatment of cells with leptomycin B, an inhibitor of exportin 1, causes actin accumulation in the nucleus (38). Secondly, the export receptor, exportin 6, appears to be responsible for the nuclear export of actin in a complex with the small ABP, profilin (39). 

Exportin 6 is a member of the importin β superfamily of transport receptors that sends actin out of the nucleus coupled with profilin (39). Although this receptor can export most types of actin isoforms (5), it is present only in higher eukaryotes (39). Chromosome region maintenance 1 ( Crm1 ), the general nuclear export receptor for cargo which has leucine-rich export signals, may be a counterpart for exportin 6 in lower eukaryotes (38). Apparently, exporting of actin coupled with actin binding proteins such as profiling is the only ligand for exportin 6 (39). This suggests that the nuclear level of actin in higher eukaryotes is specifically regulated (3). 

## Functional roles of nuclear actin

Actin is a well-known protein for its ability to shape the cytoplasm, target membrane growth, and ensure dynamic behavior of the cytoskeleton (40). In the nucleus, several regulators of actin polymerization are localized and/or translocated to the nucleus in a controlled manner, suggesting that at least some functions of actin in the nucleus are related to genome organization (15). Besides the structural role of actin in the nuclear matrix (41) actin is considered as an important factor in nuclear processes that range from chromatin remodeling to RNA splicing. 

Actin is also a component of the chromatin remodeling complex; it associates with transcription machineries and newly synthesized ribonucleoproteins (15). Recent achievements in the field of gene transcription regulation have led to conclusions that there is a vital role for actin as a chief co-factor for all three eukaryotic RNA polymerases (42). 

## Actin and nuclear matrix

The interphase nucleus is thought to contain a three-dimensional filamentous protein network referred to as the nuclear matrix which is believed to be analogous to the cell cytoskeleton. The term nuclear matrix, as first introduced by Berezney and Coffey (43) in 1974, represents a highly structured residual framework obtained from rat liver nuclei by sequential salt extractions, detergent and nuclease treatments. Further ultra-structural studies have shown that the nuclear matrix is a network consisting of branched core filaments masked with a large number of hnRNPs and regulatory proteins (44). Nuclear matrix proteins ( NMPs ) make up the internal structural framework of the nucleus and are important in maintaining spatial order within the nucleus. This scaffold is an active and dynamic structure involved in different nuclear functions. The proposed functions of the nuclear matrix are mainly related to DNA replication and/or repair, gene transcription, RNA splicing and transport processes (44,49). 

Field emission scanning electron microscopy experiments performed on *Xenopus* oocyte nuclei have clearly shown that the filamentous nuclear matrix network is basically composed of actin and protein 4.1components (50). This network may supply chromatin free channels for diffusion of ribonucleoproteins or proteins inside the nucleus. Sections of *Xenopus* oocyte nuclei analyzed by confocal microscopy also demonstrate bundles of actin filaments in the nuclear structure (5). It is suggested that these nuclear meshwork of actin filaments are capable of stabilizing the mechanical integrity of *Xenopus* oocyte nuclei (5,50). 

In the context of the presence of actin in the nuclear matrix, it is possible that actin contributes to a variety of protein associations within the nuclear framework which positions them to function as organizers of the nuclear structure. Due to the fact that the body of existing data comes mainly from the *Xenopus* oocyte model, further studies are needed to clarify the function of actin in association with the nuclear structures of somatic cells (12). 

## Actin and chromatin modification/ remodeling

Chromatin is the combination of DNA and structural/regulatory proteins in eukaryotic cells. For processes that require accession to DNA to proceed, the chromatin structure needs to be changed; this is of extreme importance in gene regulation. Chromatin modification is a key process during all genome-related processes including DNA duplication and repair, regulation of gene expression and cell differentiation. A wide variety of different multi-subunit complexes are responsible for displaying these chromatin modifications (51,52). They are capable of moving and/or replacing nucleosomes on DNA strands ( chromatin remodeler ) or mark nucleosomes with covalent modifications ( chromatin modifier ). Nucleosomes are comprised of double-stranded DNA that has complexed with small proteins called histones. Nucleosomes, which constitute the smallest units in the chromatin structure, are the chief targets in the remodeling process. They include histone core proteins around which 146 bp of DNA is wrapped and the core proteins consist of dimers of the four histone molecules H2A, H2B, H3, and H4, respectively (53). Alterations in chromatin structure can result from different interactions between DNA and histones, the process mainly regulated by chromatin remodeling and chromatin modifying enzymes (54). 

**Fig.2:**
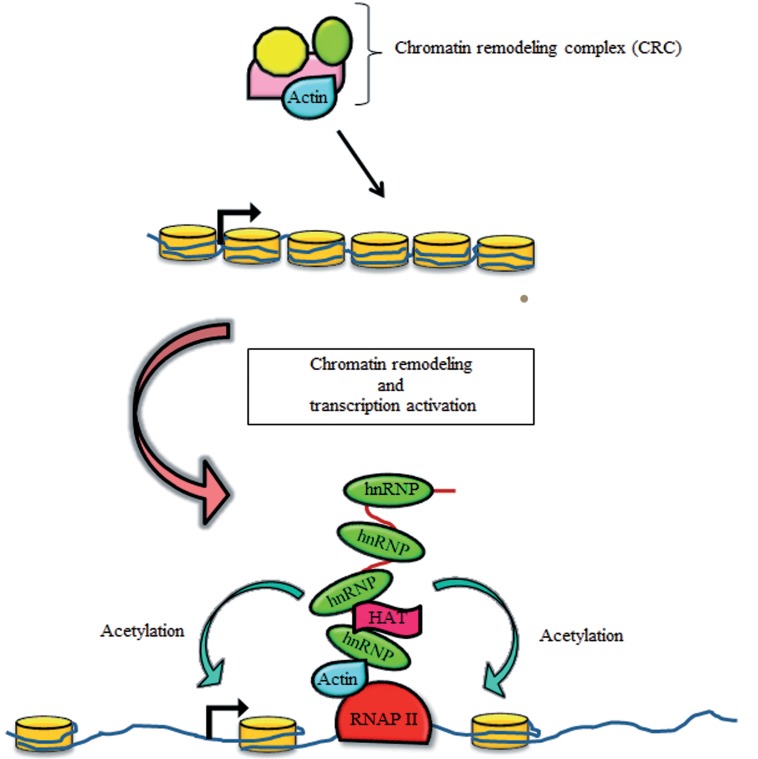
Models for the function of actin in chromatin regulation. A. Actin is a component of ATP-dependent chromatin remodeling complexes ( CRC ) involved in transcriptional activation and B. Co-transcriptional recruitment of histone modifier elements to RNA polymerase II ( RNAP II ), mediated by the presence of heterogeneous nuclear ribonucleoproteins ( hnRNPs ). During transcription, actin can be recruited to the elongating transcription machinery and facilitate recruitment of histone acetyltransferase ( HAT ) to the active gene, enhancing the processivity of RNAP II.

Recently investigators have shown that actin and actin-related proteins are involved in both chromatin remodeling and chromatin modifying complexes; this association is conserved from yeast to humans (52,54,55). It is well established that actin and a large number of actin-related proteins are vital subunits for the function of chromatin-remodeling complexes during transcriptional activation (56,59). Nuclear actin and its related proteins are also found in chromatin modifying enzymes such as histone acetyltransferase ( HAT ) complexes in a wide range of organisms that range from yeast to humans (Fig.2) (60,63). 

## Actin in association with transcription machinery

The connection of actin with transcription was first mentioned by Smith et al. (64) in 1979, when they reported that actin was co-purified with RNA polymerase II ( RNAP II ) from the slime mold P. polycephalum. Egly et al. (65) in 1984 identified actin in transcriptionally active extracts from human HeLa and calf thymus cells. A direct role of actin in transcription was reported by Scheer et al. (66) in 1984 when they showed that microinjection of antibodies directed against actin and actin-related proteins such as fragmin caused transcriptional inhibition of chromosome loops in the nuclei of amphibian oocytes in sites where active transcription occurred. Sauman and Berry (24) showed an active transcriptional role of actin in *Drosophila melanogaster* cell nuclei. They have suggested that actin recruits the RNA polymerase complex to target DNA. In 2001, actin was found in Balbiani ring localization of nascent pre-mRNA in *Chironomus* alivary gland polytene chromosomes (67). Since numerous studies have shown the functional role of nuclear actin in transcription (68,72), it is clear that actin has more than one role as an epigenetic factor involved in the process of transcription. 

## Actin in nascent transcripts

In eukaryotic cells the nascent transcripts synthesized by RNAP II undergo extensive processing steps before a functional mRNA is produced. These mRNA precursors are named heterogeneous nuclear ribonucleicacids ( hnRNAs ), a historical term based on their size, heterogeneity and cellular location (73). When hnRNAs mature into mRNAs, they associated with a variety of proteins, as a group, named hnRNPs (73,74). These proteins function in an array of cellular activities such as pre-mRNA processing, mRNA export, localization, translation and stability (75). Although actin by itself is not capable of binding to RNA, its association with hnRNPs has been reported (12). In this way actin is a component of hnRNPs in a variety of mammalian cells (30), avian erythroblasts (27), and *Xenopus* oocytes (76). 

Whereas the functional role of actin in RNP complexes is not clearly defined, studies in *Chironomustentans* have suggested that the cotranscriptional incorporation of actin into newly assembled pre-mRNPs affects chromatin architecture at transcribed genes (77). It is proposed that actin plays a role in this case via coupling with pre-mRNA-binding proteins, as a platform for the localization of a HAT activity alongside active transcription units (Fig.2). 

## Conclusion

After approximately 70 years since actin’s discovery in the cytoplasm, it is now undoubtedly accepted that actin has major roles in the cytoskeleton of eukaryotic cells and is a new member of nuclear proteins. The data mentioned in this article strongly support the idea that actin and actin-related proteins are dynamic nuclear factors involved in a multitude of nuclear functions. As discussed, nuclear actin plays functional roles in genome regulation in at least one of the three following ways: i. as a component of all three RNA polymerases in the eukaryotic cell nucleus, ii. as a constituent of nuclear ribonucleoprotein particles and iii. as a regulatory component of chromatin remodeling complexes. It is mentioned that nuclear actin can also function as a signal responsive regulator of specific transcription factors. Based on these data, a controversial issue will be the relationship between the dynamics of actin in the cytoplasm and nucleus. Due to the fact that actin in the cytoskeletal structure is very dynamic and interacts with a wide range of extracellular signals, it is suggested that actin may play a role as a sensor of extracellular signals in order to transduce signals to the chromatin state and moderate genome organization of living cells. To date, investigators suggest that as nuclear actin levels respond to different cellular stresses, it may therefore play a role in the pathology of different diseases. Also, identification of nuclear actin as a component of chromatin remodeling complexes is a current, hot topic between molecular cell biologists. Since chromatin is the critical site for maintaining the balance between gene activation and repression, scientists hope to identify potential ways by which nuclear actin can be involved in cancer. 

Together, it can be concluded that while the biological significance of nuclear actin seems to be as versatile and important as in the cytoplasm, we are only beginning to understand the mechanisms that lie behind the regulation of nuclear actin. 
